# Na-Montmorillonite Vs. Organically Modified Montmorillonite as Essential Oil Nanocarriers for Melt-Extruded Low-Density Poly-Ethylene Nanocomposite Active Packaging Films with a Controllable and Long-Life Antioxidant Activity

**DOI:** 10.3390/nano10061027

**Published:** 2020-05-27

**Authors:** Aris Giannakas

**Affiliations:** Department of Food Science and Technology, University of Patras, 30100 Agrinio, Greece; agiannakas@upatras.gr

**Keywords:** LDPE, 2-D nanomaterials, Na-montmorillonite, organo-modified-montmorillonite, thyme oil, oregano oil, basil oil, active packaging, long-life antioxidant activity

## Abstract

Nowadays, active packaging is becoming significant for the extension of the shelf life of food products via the incorporation of raw nanomaterials such as nanoclays and bioactive compounds such as essential oils (EO). This study aims to study the performance of the sodium montmorillonite (NaMt) and organically modified montmorillonite (OrgMt) as thyme (TO), oregano (OO), and basil (BO) essential oil (EO) control release nanocarriers in low-density poly-ethylene (LDPE) active films. NaMt and OrgMt nanofillers are modified with low (20 wt.%), medium (40 wt.%), and high (80 wt.%) nominal contents of TO, OO, and BO. The novel active packaging films were tested using the X-ray diffraction method (XRD), tensile, water, and oxygen barrier properties, and antioxidant activity tests. For the two most active packaging films, the lipid oxidation of chicken breast fillets estimated by the thiobarbituric-acid-reacting substances (TBARS) method. Overall study shows that both NaMtEO-based and OrgMt-based films exhibited controllable and sustained antioxidant activity. All films retained up to 50–70% of their antioxidant activity after six months of incubation. OrgMtEO-based LDPE films showed more significance applied as active packaging films than NaMtEO-based LDPE films because of their highest tensile and barrier properties.

## 1. Introduction

One of the most recent trends of nanotechnology [1] in the food industry is the incorporation of natural antioxidants (AO) to active packaging films due to its advantages compared to the addition of such natural antioxidants directly to the food [2]. Essential oils (EO) are the most widely used class of natural AO in the food packaging industry [3,4]. EO are used in packaging (1) to have an action in packaging or (2) to release into the food to avoid its oxidation. Therefore, in the first case, EO should respect the packaging legislation, and in the second case, they should respect the food legislation (community or national provisions) (European Commission 2009, Regulation (EC) 450/2009) [2].

One of the main drawbacks of direct incorporation of EO into the polymer matrix is its volatile nature, which causes rapid loss via evaporation [5,6,7]. A controlled release of EO into the polymers would be required to overcome this rapid decline of activity. It was proposed that adsorption of EO onto an inorganic porous material [8,9] could provide controlled release and protection against polymer processing conditions. In this direction, EO nanocarriers, such as commercial organically modified montmorillonite (OrgMt) [10] and halloysite [11,12], used in polymers-based [6,13,14,15] and biopolymers-based [16,17,18,19] active packaging films. Shemesh et al. [11] adsorbed carvacrol into halloysite nanotubes and developed low-density poly-ethylene (LDPE)/carvacrol/halloysite antimicrobial films. They showed [11] that the obtained antimicrobial activity of such films sustained because of the hybrid system’s slower out-diffusion of carvacrol. Recently, Campos-Requena et al. [18] studied the release of essential oil constituents from thermoplastic starch/layered silicate bionanocomposite films. They concluded that it is possible to predict the release of the EO by knowing the formulation characteristics of packaging material.

Recently, [9] presented a novel, green, and industrially applied method for the encapsulation of oregano oil (OO), thyme oil (TO), and basil oil (BO) in sodium-exchanged montmorillonite (NaMt) and commercial organically modified montmorillonite (OrgMt). The obtained NaMtEO and OrgMtEO nanohybrids are promising nanostructures for controlled-release applications. Such NaMtEO and OrgMt EO nanostructures aspire to encapsulate the most volatile fraction of EO, which is the most active one [9]. Besides, it was shown [9] that interaction mechanism between TO, OO, BO and NaMt was completely deferent as compared to the interaction mechanism between TO, OO, BO and OrgMt. In the first case, an exfoliated structure, and a strong hydrogen bonding of EO molecules on the external surface of NaMt was achieved. In the second case, an intercalated structure, and a hydrophobic interaction via the insertion of EO molecules in the OrgMt interlayer space was observed. These different mechanisms were expected to affect the release of EO molecules and the performance of such NaMtEO and OrgMtEO nanohybrids with various polymers and biopolymers matrixes.

In this study, these NaMtEO and OrgMtEO nanohybrids were applied as antioxidant nanocarriers in low-density polyethylene (LDPE)-based packaging films. NaMtEO and OrgMtEO nanostructures with low (20 wt.%), medium (40 wt.%), and high (80 wt.%) EO nominal content were used to control the obtained antioxidant activity and the possible biotoxicity of such nanohybrids. The received NaMtEO-based and OrgMtEO-based LDPE films were developed via the melt-extrusion process. The obtained films were characterized by XRD analysis. XRD is the most powerful method to prove the formation of an intercalated or an exfoliated nanostructure in polymer nanocomposite films. Tensile and water/oxygen barrier properties, which are crucial for such packaging films, were also studied. Antioxidant activity of all obtained films were investigated according to the di(phenyl)-(2,4,6-trinitrophenyl) iminoazanium (DPPH) oxidation method. Antioxidant activity investigation was used to compare the activity of NaMtEO-based and OrgMtEO-based LDPE films and the activity of TO-, OO-, and BO-based films. One of the NaMtEO-based and one of the OrgMt-based LDPE films with the highest antioxidant activity were tested as active packaging films for vacuum-packaged chicken breast fillets and the lipid oxidation value was determined. All experiments were designed to compare the sodium montmorillonite (NaMt) and organically modified montmorillonite (OrgMt) as thyme (TO), oregano (OO), and basil (BO) essential oil (EO) nanocarriers and as nano-reinforcements in the development of LDPE active films.

## 2. Materials and Methods

### 2.1. Materials

#### 2.1.1. Essential Oil Used

Origanum, thyme, and basil EO were purchased from Esperis s.p.a., Via A. Binda, 29, 20143 Milano (Italia). According to safety data sheets, the % mass composition of oregano oil was 60–carvacrol, 10–12.5% thymol, 10–12.5% paracymene, 5–7% alpha-pinene, 5–7% 1-isopropyl-4-methyl-1,4-cyclohexadiene p-mentha-1,4-diene, and 1–3% terpinene-4-olo, beta-myrcene and (R)-p-mentha-1,8-diene. Thyme oil consisted of 50–60% thymol, 15–20% para-cymene, 10–12.5% 1-isopropyl-4-methyl-1,4-cyclohexadiene p-mentha-1,4-diene, 3–5% carvacrol, and 1–3% linalool, beta-caryophyllene, beta-myrcene, (R)-p-mentha-1,8-diene, alpha-pinene, borneol and terpinene-4-olo. Basil oil consisted of 70–80% estragole, 7.5–10% linalool, 1–3% eucalyptol, 0.5–1.0% eugenol, and 0.5–1.0% D-limonene.

#### 2.1.2. Nanoclay Used

(1) Sodium-exchanged montmorillonite (NaMt) with code name Nanomer^®^ PGV with mass density 2.6 g/cm^3^ and Cation Exchange Capacity (CEC) value 145 milliequivalent (meq)/100 g produced by Nanocor Inc. (Hoffman Estates, IL, USA), and supplied by Sigma-Aldrich (St. Louis, MO, USA). The chemical composition of NaMt was 62.9% SiO_2_, 19.6% Al_2_O_3_, 3.35% Fe_2_O_3_, 3.05% MgO, 1.68% CaO, and 1.53% Na_2_O. (2) Commercial, organically modified montmorillonite (OrgMt) NANOMER^®^-I·44P produced by Nanocor Inc. (Hoffman Estates, IL, USA), and supplied by Sigma-Aldrich (St. Louis, MO, USA). NANOMER^®^-I.44P is an -onium ion-modified clay containing ~40 wt.% dimethyl dialkyl (C14–18) organic ammonium modifier.

### 2.2. Methods

#### 2.2.1. Preparation of NaMtEO and OrgMtEO Nanohybrids

The NaMtEO and OrgMtEO nanohybrids were prepared as previously reported [9]. Briefly, 5 g of each clay were spread in an aluminum beaker. In the middle of an aluminum beaker, a smaller quartz beaker was placed and filled with the appropriate quantity of each EO. The amount of EO used was 1.0, 2.0, and 4.0 g to achieve a final nominal composition of EO to nanoclays 20, 40, and 80 wt.%, respectively. Then, the aluminum beaker was sealed and put in an oven at 120 °C for 24 h. Under these conditions, the most volatile EO components evaporated and adsorbed into NaMt and OrgMt. Thus, NaMt and OrgMt nanohybrids with low (20 wt.%), medium (40 wt.%), and high (80 wt.%) EO nominal content were obtained. The obtained nanohybrids were labeled as NaMtEOx and OrgMtEOx. EO were labeled as TO, OO, and BO when thyme oil, oregano oil, and basil oil correspondingly were used while x was the nominal composition of EO used to modify clays and took the values 20, 40, 80.

#### 2.2.2. Preparation of LDPENaMtEO and LDPEOrgMtEO Films

The LDPENaMtEO and LDPEOrgMtEO films were prepared by the melt-mixing process. For the preparation, a minilab twin extruder co-rotating (Haake Mini Lab II, ThermoScientific, ANTISEL, S.A., Athens, Greece) was used. For minimal loss of the EO substance during the melt processing, the uniform temperature operated was 140 °C at a screw speed of 100 rpm for 5 min total time processing. The nominal composition of NaMtEO and OrgMtEO nanohybrids were added to LDPE fixed to 3 wt.%. According to Thernogravimetric (TG) experiments reported previously [9], EO content for low (20 wt.%), medium (40 wt.%), and high (80 wt.%) loaded NaMtEO and OrgMtEO nanohybrids was approximately 5, 10, and 15 wt.%, correspondingly. Thus, the final EO content of the obtained films was approximately 0.15 wt.% for LDPENaMtEO20 and LDPEOrgMtEO20 samples, 0.30 wt.% for LDPENaMtEO40 and LDPEOrgMtEO40 samples, and 0.45 wt.% for LDPENaMtEO80 and LDPEOrgMtEO80 samples, correspondingly. In a typical procedure, 4.85 g of LDPE pellets and 0.15 g each of NaMtEO and OrgMtEO nanohybrids were physically mixed in a glass beaker and added to the extruder at the same upstream feed port. The obtained melt compound strands were cut to small granules with a granulating machine. Finally, films were produced with approx. 10-cm diameter by hot-pressing approximately 1 g of obtained granules at 110 °C under 2.5 megapaschal (MPa) constant pressure for 3 min, using a hydraulic press with heated platens. “Blank” samples, by mixing LDPE with commercial NaMt and OrgMt, were also prepared for comparison.

#### 2.2.3. X-Ray Diffraction (XRD)

The XRD measurements of the films were performed on a Brüker D8 Advance diffractometer (Bruker, Analytical Instruments, S.A., Athens, Greece) pattern at low angle using with a LINXEYE XE high-resolution energy dispersive detector. The diffractometer was thermostated at 20 °C, and the beam monochromator was operated at a voltage of 40 kV and a beam current of 40 milliampere (mA). CuK(alpha) radiator worked in 1-D mode with a wavelength λ = 1.541874 Å. Scanning parameters were set as follows: Two theta ranges, 2.5°–25° for NaMt-based films and 0.5°–25° for OrgMtEO-based films, increment 0.03°, Position Sensitive Detectors (PSD) 0.764, counting time 1022 s, and slit width 0.6 mm. The characterization of the nanoclay-containing samples was based on the 001 reflection peak.

#### 2.2.4. Tensile Properties

Tensile measurements were carried out on all prepared nanostructured films, according to the American Society for Testing and Materials (ASTM) D638 method, using a Simantzü AX-G 5kNt instrument (Simantzu. Asteriadis, S.A., Athens, Greece). Three to five samples of each film were tensioned at an across-head speed of 10 mm/min. The samples were dumbbell-shaped samples with gauge dimensions of 10 mm × 3 mm × 0.22 mm. Force (N) and deformation (mm) were recorded during the test. Based on these data and the gauge dimensions, stress, stain, and modulus of elasticity were also calculated.

#### 2.2.5. Water Vapor Transmission Rate (WVTR)

For WVTR measurement, films with an average diameter of 2.5 cm and an average thickness around 100 μm were produced by using the applicable ring of hydraulic press and by hot-pressing approximately 0.2 g of obtained granules at 110 °C under 2 MPa constant pressure. WVTR of all nanocomposite films were determined at 38 °C using the apparatus and methodology described in the ASTM E96/E 96M-05 and previous reports [20,21,22]. Films with approx. 2.5 cm in diameter and around 100-μm average thickness were sealed by a rubber O-ring on top of Plexiglas test bottles containing dried silica gel and placed in a glass desiccator with a 200 mL saturated magnesium nitrate solution (50% relative humidity (RH)). Test bottles were weighed periodically for 24 h, and the WVTR was calculated according to the following equation:

WVTR = (G/t)/A(1)

G is the weight gain of the tested bottles in g, t is the time in hours, G/t is the slope of the straight line in the diagram of ΔG as a function of time (f(t)), and A is the permeation area. The weight of the tested films was measured before and after the WVTR test to exclude any absorption phenomena.

#### 2.2.6. Oxygen Transmission Rate (OTR) Measurements 

For OTR measurement, films with average diameter of 10 cm and average thickness approximately 350 to 400 μm were produced by hot-pressing approximately 1.8 g of obtained granules at 110 °C under 2 MPa constant pressure. Oxygen transmission rate (OTR) was measured using an 8001, Systech Illinois Instruments Co., Johnsburg, IL, USA according to Standard Method D 3985-81 (ASTM 1989). The instrument’s measurement range was 0.008–432,000 cc/m^2^/day, and testing was performed at 23 °C at 0% RH. Oxygen transmission rate (OTR) was obtained in cc O_2_/m^2^/day. Three measurements were performed for each tested sample. The oxygen permeability (OP) values of the tested samples were calculated by multiplying the OTR values with the average film thickness.

#### 2.2.7. Antioxidant Activity Evaluation of Films

Evaluation of the antioxidant activity of each film was investigated via the determination of di(phenyl)-(2,4,6-trinitrophenyl) iminoazanium (DPPH) radical scavenging activity. Firstly, 300 mg of each film was cut into small pieces (approx. 5 mm × 5 mm) and placed in dark-colored glass bottles with a plastic screw cap. Then, the glass bottles were filled with 10 mL of 20 ppm (mg/L) ethanolic DPPH solution and incubated at 25 °C for 24 h, 48 h, 72 h, and 96 h in darkness. The percent of antioxidant activity of films after 24 h, 48 h, 72 h, and 96 h incubation was calculated by measuring the absorbance (Abs) at 517 nm in a Jasco V-530 photometer (Asteriadis, S.A., Athens, Greece) according to the equation:

% Antioxidant activity = (Abs_control_ − Abs_sample_)/Abs_control_) × 100 (2)

The DPPH solution without extract solution was used as control.

To evaluate the long-life antioxidant activity of LDPENaMtEO and LDPEOrgMtEO films, a series of films were incubated under 50 RH% and 25 °C for six months. Subsequently, the antioxidant activity of such films was also tested. For this experiment, the same experimental conditions were used (300 mg film/20 ppm initial ethanolic DPPH solution), and antioxidant activity was determined by measuring the absorbance at 517 nm after 24 h, according to Equation (2).

#### 2.2.8. Lipid Oxidation

##### Sample Preparation

**Figure 1:**
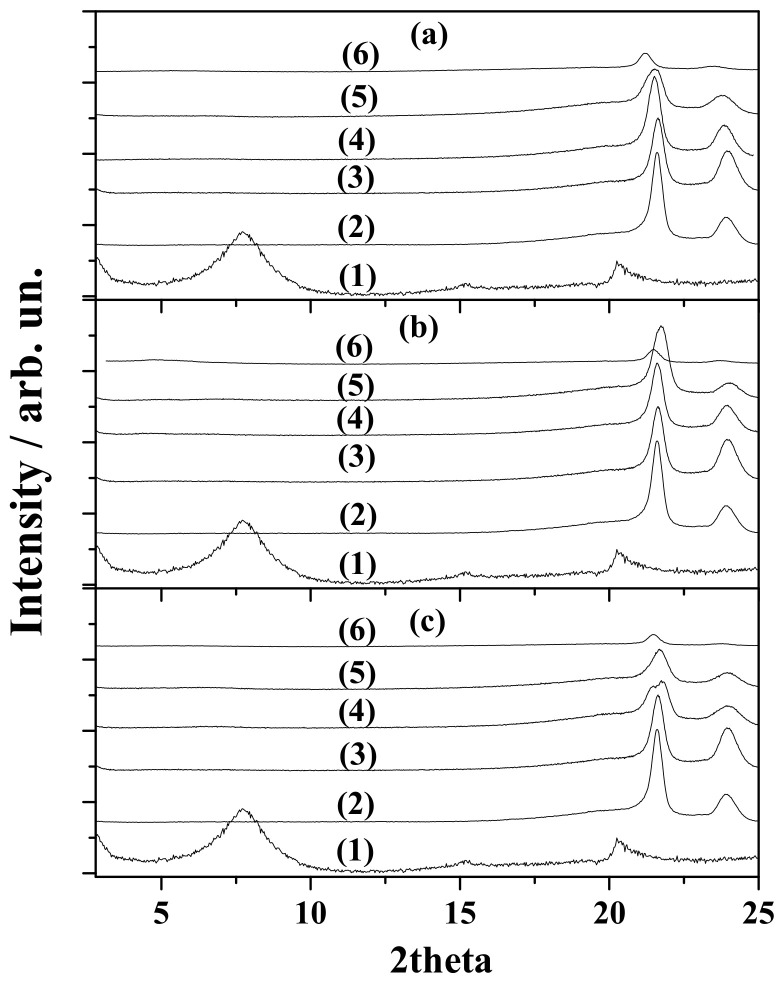
XRD patterns of (**a**) LDPENaMtTO, (**b**) LDPENaMtOO, and (**c**) LDPENaMtBO films. (1) NaMt as received, (2) pure LDPE film, (3) “blank” LDPENaMt film, (4) LDPENaMtEO20 film, (5) LDPENaMtEO40 film, and (6) LDPENaMtEO80 film.

Within two hours after the slaughter, three freshly skinned and deboned chicken breasts with approximate weight 200 g were provided by a local poultry processing plant (Pindos S.A., Ioannina, Greece) in insulated polystyrene boxes on ice. Chicken meat fillets were aseptically portioned into smaller pieces, approx. 20 g each. Each small chicken piece was immediately wrapped. For each piece’s packaging, two disk-shaped films were used with a 10-cm diameter and 0.06-mm average thickness. The LDPENaMtTO80 and LDPEOrgMtTO80 nanocomposite films, which were those that exhibited the highest antioxidant activity, were chosen for lipid oxidation experiments. As a “blank” sample, pure LDPE disk-shaped films were operated. The two disk-shaped films (10-cm diameter, 100-μm thickness) of each sample were put inside a commercial polyethylene (PE) vacuum-packaging bag. In total, three samples of each tested film were found (see Figure 1) to have a reliable statistical analysis of results. All utensils used, including the LDPE disk-shaped films and PE vacuum packaging bags, were sanitized with ethanol to avoid cross-contamination. Τhe samples were vacuum-packed with a vacuum sealer, SFS 120 A1 machine, and stored for 10 days at 4 °C until analyzed by the Thiobarbituric acid reactive substances (TBARS) method to determine the degree of lipid oxidation suffered.

##### TBARS Method

The lipid oxidation of chicken breast fillets was estimated by the thiobarbituric-acid-reacting substances (TBARS) assay, as outlined by Panea et al. (2014) [23] with small modifications and expressed as mg malonaldehyde (MDA)/kg of chicken breast fillets. Briefly, meat samples were mixed with trichloroacetic acid and centrifuged. The supernatant was removed. The filtrate was vortexed with thiobarbituric acid, homogenized, and incubated at 97 °C for 20 min in a water bath. The absorbance at 532 nm was then measured. A standard calibration curve was created with increasing concentrations of 1,1,3,3, tetra methoxy propane (99%), the precursor of malonaldehyde (MDA). The final conversion of 1,1,3,3 tetramethoxypropane to MDA was accomplished by multiplying the number of l M (Molarity = mol/L) of 1,1,3,3 tetramethoxypropane equivalent per gram of sample by the molecular weight of MDA.

### 2.3. Statistical Analysis

All measurements were carried out at least in triplicate for each sample. The statistical analysis was performed using the Statistical Package SPSS 25 for windows (SPSS Inc., Chicago, IL, USA). The results of this analysis are presented below in the next section.

## 3. Results

### 3.1. XRD

Figure 1 shows the XRD plots of all LDPENaMtTO (Figure 1a), LDPENaMtOO (Figure 1b), and LDPENaMtBO (Figure 1c) samples. Figure 1 also shows the plots of the “blank” LDPENaMt film, the pure LDPE film, and the NaMt as-received sample for comparison. In the XRD plot of NaMt as-received sample (see the line (1) in Figure 1), the characteristic peak of montmorillonite at around 7.3° was recorded. This peak in XRD plots of all LDPENaMtEO samples, as well as in the XRD plot of “blank” LDPENaMt sample, almost disappeared. So, in all LDPENaMtEO samples, an exfoliated structure was favored. This result should have been expected, according to our previous report [9]. It proved [9] that EO molecules were adsorbed via a hydrogen bond on the external surface of NaMt layers and did not insert into the interlayer NaMt space. So, the interlayer galleries of NaMtEO nanohybrids remained hydrophilic and did not favor the insertion of LDPE’s hydrophobic chains. In the XRD plot of pure LDPE film (see the line 2 plots in Figure 1), the characteristic peaks at around 21.8° and 24.0° corresponded to the partly crystalline and the partially amorphous structure of LDPE. In the XRD plots of all LDPENaMtEO films, these LDPE’s characteristic peaks decreased and slightly shifted in lower angles as the % content of EO increased. Both observations suggest that by increasing modification of NaMt external surface with EO molecules, the interplay of NaMtEO platelets with LDPE chains was supported.

**Figure 2:**
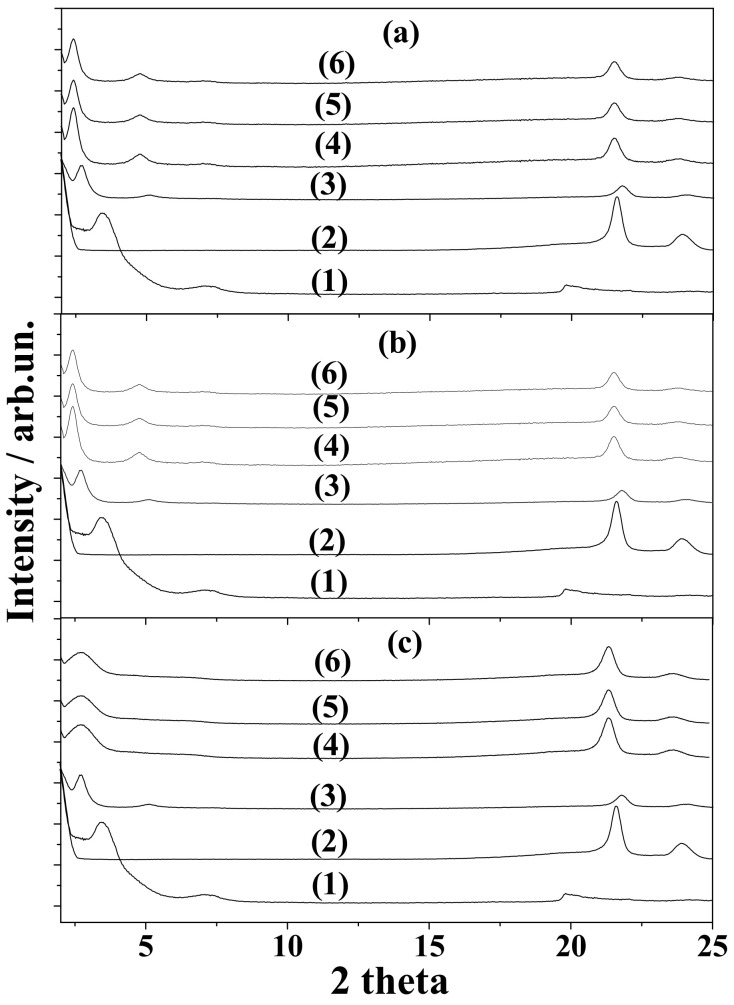
XRD patterns of (**a**) LDPEOrgMtTO, (**b**) LDPEOrgMtOO, and (**c**) LDPEOrgMtBO films. (1) OrgMt as-received, (2) pure LDPE film, (3) “blank” LDPEOrgMt film, (4) LDPEOrgMtEO20 film, (5) LDPEOrgMtEO40 film. and (6) LDPEOrgMtEO80 film.

Figure 2 shows the XRD plots of all LDPEOrgMtTO (Figure 2a), LDPEOrgMtOO (Figure 2b), and LDPEOrgMtBO (Figure 2c) samples. Figure 2 also shows the XRD plots of the “blank” LDPEOrgMt film, the pure LDPE film, and the pure OrgMt sample for comparison. In all graphs, a clear shift of 001 OrgMt basal space reflection from approximately 3.5° for the OrgMt as-received sample to approximately 2.5–2.7° for all LDPEOrgMtEO nanocomposite films was recorded. In advance, the LDPE’s characteristic peaks at around 21.8° and 24.0° decreased for all LDPEOrgMtEO nanocomposite films. These two observations implied the insertion of the LDPE chains inside the interlayer space of OrgMtEO nanostructures. Table 1 lists the calculated d_001_ values (d-spacing values of 001 reflection) for comparison. It was found that the interlayer space of the OrgMt sample increased to around 36 Å for both OrgMtTO- and OrgMtOO-based LDPE films and to approximately 33 Å for OrgMtBO-based LDPE films. These values are higher than 32 Å, which was the corresponding d_001_ value for the “blank” LDPEOrgMt sample (see Table 1). So, the incorporation of EO molecules in the interlayer space of OrgMt [9] supported the slit of LDPE chains into the OrgMt galleries. So, from XRD plots (Figure 2) and the d_001_ values in Table 2, I concluded the formation of an intercalated nanocomposite structure for all LDPEOrgMtEO films. The intercalated structure seemed to be “stronger” for all LDPEOrgMtEO samples than for the LDPEOrgMt “blank” sample.

**Figure 3:**
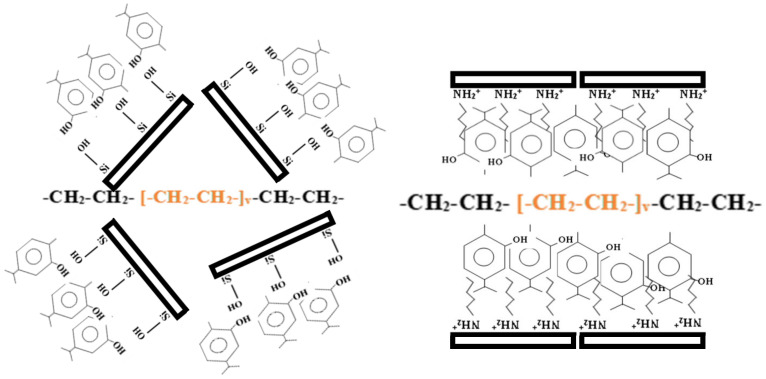
Schematic illustration of the exfoliated structure of LDPENaMtEO samples (left part) and the intercalated structure of LDPEOrgMtEO sample (right part).

Thus, as illustrated in Figure 3, XRD results showed an exfoliated structure for all LDPENaMtEO films and an intercalated structure for all LDPEOrgMtEO films. The intercalated nanocomposite structure was favorable for the tensile and barrier properties of packaging films.

### 3.2. Tensile Properties

**Figure 4:**
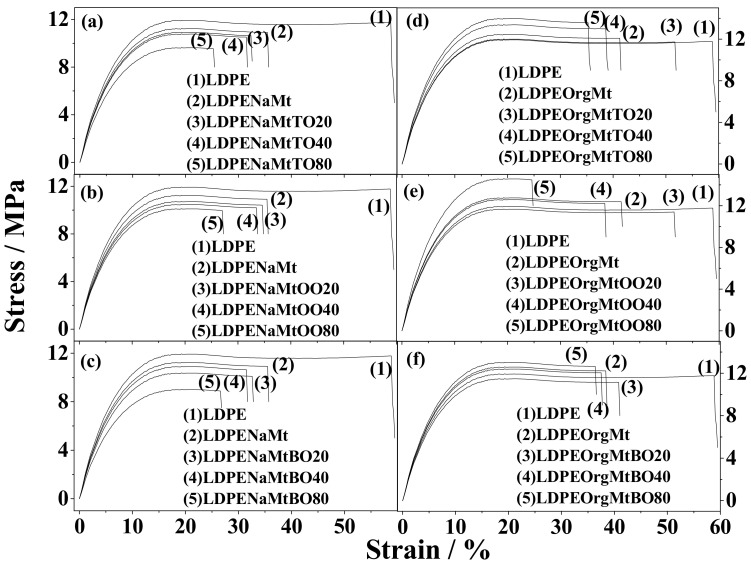
Stress-strain curves of all tested LDPENaMtEO (left part) and LDPEOrgMt films.(**a**) LDPENaMtTO films, (**b**) LDPENaMtOO films, (**c**) LDPENaMtBO films, (**d**) LDPEOrgMtTO films, (**e**) LDPEOrgMtOO films and (**f**) LDPEOrgMtBO films.

A food packaging film is required to maintain film integrity to withstand the stress that occurs during shipping, handling, and storage. Figure 4 shows the characteristic strain-stress curves for all tested LDPENaMtEO films (left part) and LDPEOrgMtEO films (right part) as well as for the pure LDPE and the “blank” LDPENaMt films for comparison. Table 1 provides the average values of the E Modulus, ultimate tensile strength (σ_uts_), and % strain at break (% ε) of all tested samples for comparison.

The strain-stress curves (Figure 4) and the calculated values in Table 1 show that all LDPENaMtEO samples, as well as LDPENaMt “blank” sample, presented lower Young’s Modulus, tensile strength, and % ε values than the pure LDPE sample did. This result should be expected for such exfoliated samples.

On the contrary, (see Figure 4 and Table 1) all LDPEOrgMtEO samples, as well as the “blank” LDPEOrgMt sample, presented higher Young’s Modulus and tensile strength values than pure LDPE film. This result should be expected for nanocomposites with an intercalated structure. More specifically, OrgMt addition led to an approx. 10% enhancement of stiffness (Ε) and an approx. 6% enhancement of tensile strength (σ_uts_) of the “blank” LDPEOrgMt film as compared to the pure LDPE film. Besides, the % ε value of the LDPEOrgMt sample was approximately 45% lower than the % ε value of the pure LDPE film. In advance, for LDPEOrgMtEO samples with low (20%) and medium (40%) EO nominal content, stiffness (Ε), and strength (σ_uts_) values were equal or slightly higher as compared to the stiffness (Ε) and the strength (σ_uts_) values of the “blank” LDPEOrgMt sample. At the same time, % ε values further decreased. For LDPEOrgMtEO samples with high (80%) EO nominal content, higher values of stiffness (Ε) and strength (σ_uts_) were obtained as compared to the LDPEOrgMt sample. This result supports our recent report [9]. It showed that for high 80 wt.% EO loadings on OrgMt clay, an optimum bilayer orientation of EO molecules took place in OrgMtEO nanohybrids [9]. Thus, for the LDPEOrgMtOO80 sample, the stiffness and the tensile strength values were approx. 28% and 20% higher than the corresponding stiffness and strength values of the pure LDPE film.

### 3.3. Barrier Properties

Water vapor and oxygen are two of the main permeants studied in food packaging applications. They may transfer from the internal or external environment through the polymer package wall, resulting in a continuous change in product quality and shelf life. The proper barrier to moisture and oxygen in food systems can increase the quality of the product. Table 2 lists the calculated WVTR and OP values for all tested LDPENaMtEO, LDPEOrgMtEO films, as well as for the pure LDPE film and the “blank” LDPENaMt and LDPEOrgMt films, for comparison. For the LDPENaMtEO samples, higher WVTR and OP values were observed as compared to WVTR and OP values of the pure LDPE film. For LDPENaMtEO films with low (20%) and medium (40%) EO content, lower WVTR and OP values were observed than the corresponding WVTR and OP values of the LDPENaMt film. For the LDPENaMtEO films with high (80%) EO content, WVTR and OP values were equal or higher than the corresponding WVTR and OP values of the “blank” LDPENaMt film. This result supported XRD results, where it was concluded that NaMt’s and NaMtEO’s addition to LDPE led to the exfoliated structure.

Moreover, all OrgMt-based LDPE films showed lower WVTR and OTR values than pure LDPE film (Table 2). Besides, by increasing EO content, water and oxygen barrier properties increased further. The increase in barrier properties of LDPE nanocomposite films modified with organic- modified clays is well known [24,25,26,27,28]. Herein, it showed that the extra modification of OrgMt with EO molecules further improved water and oxygen barrier properties. Thus, LDPEOrgMtOO80 film achieved 32% and 18% lower WVTR value than pure LDPE and “blank” LDPEOrgMt samples, correspondingly, while its OTR value was 75% and 66% lower than the corresponding OTR values of LDPE and “blank” LDPEOrgMt samples.

### 3.4. Antioxidant Activity of Films

**Figure 5:**
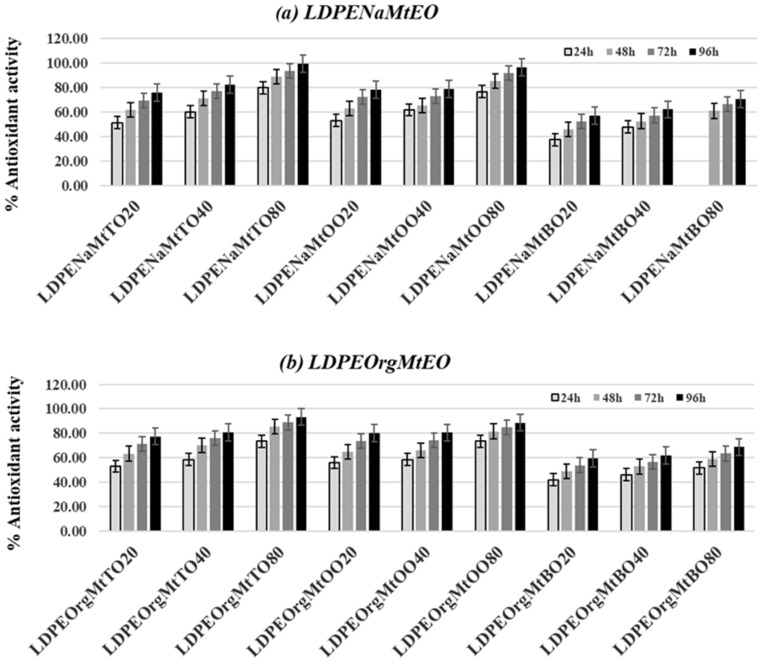
(**a**) column bar diagram of % antioxidant activity values of all tested LDPENaMtEO films after 24, 48, 72, and 96 h and (**b**) column bar diagram of % antioxidant activity values of all tested LDPEOrgMtEO films after 24, 48, 72, and 96 h.

DPPH radical scavenging activity is one of the most frequently used methods to predict the antioxidant activity of packaging films. Figure 5 depicts the % antioxidant activity values of all tested LDPENaMtEO (Figure 5a) and LDPEOrgMtEO films (Figure 5b). Besides, Table 2 includes % antioxidant activity values after 24 h for all tested films for comparison. For all samples, it was obtained that antioxidant activity increased continuously during the four days of the experiment. Thus, the loading of EO molecules on NaMt and OrgMt clay substrates succeeded in controlling EO’s release rate. From the calculated DPPH values of Table 2, it resulted:

(1) By increasing EO content, the antioxidant activity of all tested films further increased.
(2) NaMtTO-, NaMtOO-, OrgMtTO-, and OrgMtOO-based films exhibited much higher antioxidant activity than NaMtBO- and OrgMtBO-based films.
(3) Almost equal antioxidant activity values were recorded for both LDPENaMtEO and LDPEOrgMtEO films.

The increase of antioxidant activity of tested films by increasing the EO nominal content validated the preparation procedure followed. So, the use of NaMtEO and OrgMtEO hybrids’ nanocarriers loaded with different amounts of TO, OO, and BO allowed the control of antioxidant activity of obtained films.

The higher antioxidant activity of TO- and OO-based films, than the antioxidant activity of BO-based films, was caused by the different components of such EO. According to datasheets, TO consisted of 50–60 wt.% thymol and OO consisted of 60–70 wt.% carvacrol and 10–20 wt.% thymol while BO consisted of 70–80 wt.% estragole. Thymol and carvacrol are known [3,29,30] to have high antioxidant and antimicrobial activity, which is why they were reported [11,31,32,33,34] to use in active packaging films.

Finally, as denoted above, both the LDPENaMtEO and the LDPEOrgMtEO films exhibited, in general, similar antioxidant activities. This result was because of the equal final amount of EOs absorbed in NaMtEO and OrgMtEO, as proved previously by TG experiments [9]. Thus, the recorded antioxidant activity of obtained films varied according to the EO amount loaded on the NaMtEO and OrgMt nanohybrids.

**Figure 6:**
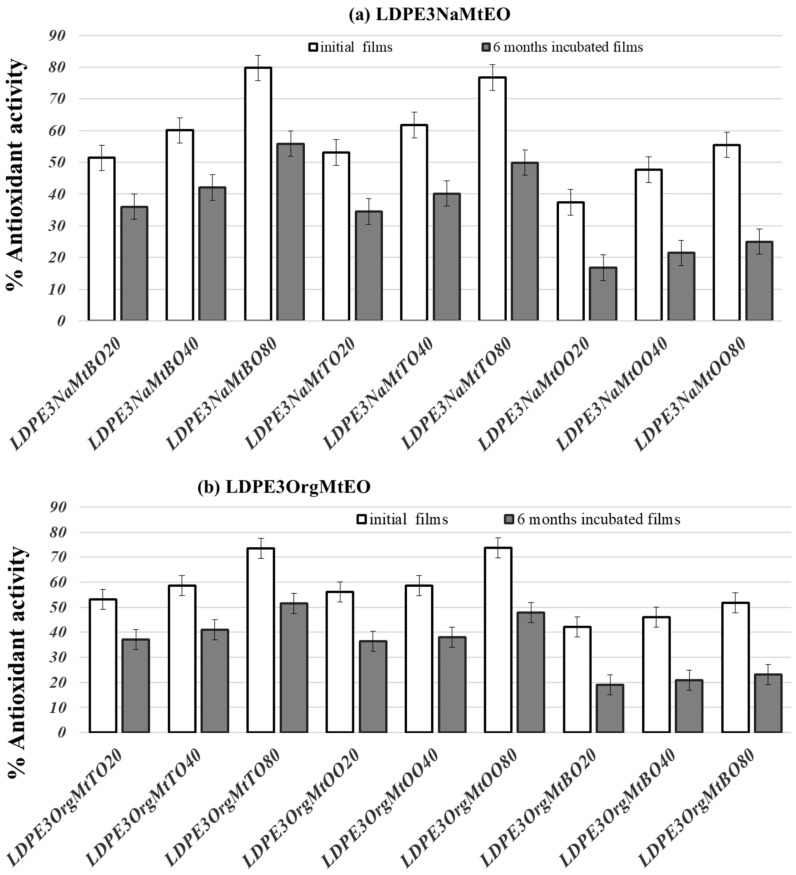
Comparison column bar diagram of initial and after six months % antioxidant activity values of all (**a**) LDPENaMtEO and (**b**) LDPEOrgMtEO films.

Figure 6 compares the antioxidant activity values of original films with the antioxidant activity values after six months of incubation of films. For all LDPENaMtEO and LDPEOrgMtEO, antioxidant activity values after six months’ incubation were approx. 30 to 50% lower than the antioxidant activity values of original films. Nonetheless, all films retained a significant fraction of their antioxidant capacity. More specifically, after four days TO- and OO-containing films exhibited an antioxidant capacity of over 50%. This result validated the use of such films as active packaging films for long-life food preservation. The sustained, long-life antioxidant activity of both LDPENaMtEO and LDPEOrgMtEO films was consistent with the kind of bonding/encapsulation of TO, OO, and BO molecules. As shown previously [9] and illustrated in Figure 3, EO molecules strongly bonded via a hydrogen bond on the external surface of NaMt and encapsulated in the interlayer space of OrgMt. Thus, the intense bonding in the case of NaMtEO samples and the in-depth encapsulation in the case of OrgMtEO samples led to obtaining LDPENaMtEO and LDPEOrgMtEO films with sustained antioxidant activity.

### 3.5. Lipid Oxidation

**Figure 7:**
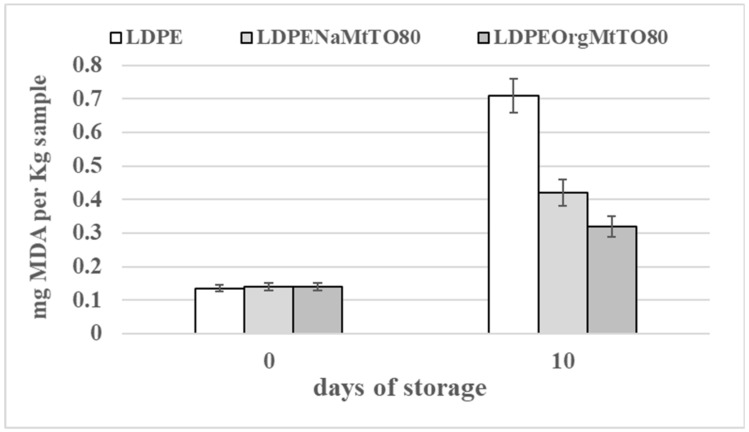
Column bar diagram of Thiobarbituric acid reactive substances (TBARS’) values of chicken breast fillets vacuum packaged with LDPE, LDPENaMtTO80, and LDPEOrgMtTO80 films after 0 and 10 days of storage at 4 °C.

Lipid oxidation is a major contributor to flavor and quality deterioration in meat and meat products. Figure 7 presents the effect of the most active LDPENaMtTO80 and LDPEOrgMtTO80 films on the lipid oxidation stability of chicken breast fillets during chilled storage under vacuum conditions in comparison with the LDPE packaging film used as a “blank” sample. The initial TBARS’ values of chicken breast fillets were approx. 0.14 mg MDA/kg. These values followed data reported elsewhere for fresh chicken breast fillets [35,36].

Moreover, Thiobarbituric acid reactive substances (TBARS) values for all chicken meat treatments after 10 days of vacuum storage varied between 0.32 and 0.71 mg. MDA/kg meat. These values were well below the 2 mg/kg threshold at which rancid off-flavors become noticeable [37]. More specifically, it was recorded that both LDPENaMtTO80 and LDPEOrgMtTO80 samples achieved much lower lipid oxidation values than LDPE classic film. The most moderate TBA values were recorded for the LDPEOrgMtTO80 film. This result probably suggests a correlation with the highest water and oxygen barrier properties marked here above for LDPEOrgMtEO samples compared to water and oxygen barrier of LDPENaMtEO samples.

### 3.6. Statistical Analysis

Experimental data of tensile, barrier, and antioxidant properties were processed using the statistical software SPSS ver. 25. In more detail, for all statistical tests, it was assumed a confidence interval of C.I. = 95%, which is the most common value used for such analyses. Thus, the value of the statistical significance level was a = 0.05. Results for mean values and standard deviation were included in Table 1 for E, σ_uts_, % ε values, and in Table 2 for WVTR, OP, and % Antioxidant Activity values, correspondingly. Besides, for each one parameter, the hypothesis H0:(mean values could assume as equal) was examined for all possible coupling combinations of film samples. By this way, it supported the hypothesis that every parameter had a statistically different mean value considering different samples. The normality tests for datasets indicated that some of them could not assume as normal distributions. Also, according to Levene’s criterion, variance homogeneity tests showed that it did not obtain homogeneity in some films. Thus, the ANOVA method was used for the testing of means’ equality. Instead, we used the non-parametric Kruskal–Wallis method. Table 3 presents the results.

The Sig. value (significant value) of Table 3 was compared with the significance level (a). From Table 3, it is evident that, in all cases, the Sig. values were smaller than the significance level values (a). Thus, in all cases and for all parameters, mean values were statistically different. The smaller the Sig. value compared to significance level values (a), the more assured it was that mean values were statistically unequal. For Sig. = 0, the inequality of means was 100% statistically assured. For Sig. close to (a) values, the inequality of means was limitedly assured. According to the clarifications mentioned above, the empirical Equation (3) was developed for the calculation of an empirical factor we call “inequality assurance” (IA). This factor is the percentage of the deviation of Sig. from (a) values towards 0. The calculation of the “inequality assurance” (IA) is calculated as follows:

IA = (a − Sig.)/a × 100(3)

From Table 3, it is evident that in all cases, the mean values’ inequality ranged from 90% to 98% and was statistically assured firmly.

## 4. Conclusions

The main conclusion of this study was the successful incorporation of novel NaMtEO and OrgMtEO nanohybrids into the LDPE matrix to develop promising active packaging films with controllable and long-life antioxidant activity. The main thrust of innovation of the preparation procedure presented here was based on using both NaMtEO and OrgMtEO nanohybrids loaded with low, medium, and high contents of TO, OO, and BO, thus controlling the antioxidant activity of the developed active packaging films. All NaMtEO- and OrgMtEO-based active films achieved significant, controllable, and sustained antioxidant activity. The highest antioxidant activity was for films containing TO and OO. Moreover, for OrgMtEO-based LDPE active packaging films, an intercalated nanocomposite structure was achieved. Thus, superior tensile and barrier properties, as compared to NaMtEO-based LDPE active packaging films, was observed. LDPENaMtTO80 and LDPEOrgMtTO80 films were chosen as two of the most antioxidant active films and were tested for lipid oxidation values of chicken breast fillet vacuum packaged at 4 °C. The obtained lipid oxidation values for LDPENaMtTO80 and LDPEOrgMtTO80 active films after 10 days of storage were 41% and 55% lower, correspondingly, as compared to the lipid oxidation value of the pure LDPE film. Overall, the OrgMtEO-based LDPE films were the most promising candidates as active packaging films. Besides, both TO- and OO-based films were the most valuable to use in the future for food processing. Although LDPENaMtEO-based films resulted in reduced mechanical and barrier properties, their controllable and sustained antioxidant activity creates the prospect that such NaMtEO nanocarriers could be promising nanofillers for grafted polymers- or biopolymers-based active packaging films. Biotoxicity, migration, and antimicrobial studies of all active films obtained here are ongoing and will be the subject of the next study.

## Figures and Tables

**Table 1:** The d_001_ spacing values along with the Young’s Modulus E, the ultimate strength σ_(uts),_ and the % elongation at break % ε_b_ mean (standard deviation) values of all LDPENaMtEO and LDPEOrgMtEO nanocomposite films as well as of the LDPENaMt, the LDPEOrgMt, and of the pure LDPE film.

Sample Code Name	d_001_/(Å)	Young’s Modulus E (St.Dev.) (MPa)	σ_(uts)_ (St.Dev.)(MPa)	%ε_b_ (St.Dev.)
LDPE		175.3 (10.1)	11.7 (0.5)	58.5 (4.5)
LDPENaMt	n.p.	165.1 (8.9)	11.0 (0.5)	35.5 (3.5)
LDPENaMtTO20	n.p.	160.5 (12.5)	10.6 (0.8)	32.4 (2.3)
LDPENaMtTO40	n.p.	158.4 (10.5)	10.5 (0.7)	31.6 (2.5)
LDPENaMtTO80	n.p.	141.5 (10.2)	9.5 (0.6)	25.5 (3.2)
LDPENaMtOO20	n.p.	157.5 (16.2)	10.9 (0.8)	34.4 (4.2)
LDPENaMtOO40	n.p.	153.4 (14.8)	10.4 (0.9)	33.6 (4.1)
LDPENaMtOO80	n.p.	131.2 (10.8)	10.2 (0.7)	27.5 (2.8)
LDPENaMtBO20	n.p.	160.2 (17.8)	10.0 (0.8)	32.4 (3.5)
LDPENaMtBO40	n.p.	152.3 (15.9)	10.7 (0.8)	32.6 (3.4)
LDPENaMtBO80	n.p.	132.3 (13.5)	8.9 (0.7)	26.5 (2.7)
LDPEOrgMt	32.0	195.0 (10.4)	12.4 (0.6)	38.3 (3.8)
LDPEOrgMtTO20	36.1	170.3 (12.2)	11.6 (0.6)	51.7 (5.6)
LDPEOrgMtTO40	36.2	185.8 (13.4)	12.3 (0.7)	41.3 (4.8)
LDPEOrgMtTO80	36.8	218.3 (9.5)	13.6 (0.7)	35.6 (3.9)
LDPEOrgMtOO20	36.5	171.5 (14.5)	11.3 (0.7)	39.6 (4.1)
LDPEOrgMtOO40	36.5	187.4 (14.2)	12.3 (0.7)	38.5 (3.8)
LDPEOrgMtOO80	36.8	224.2 (11.5)	14.5 (0.5)	34.5 (3.5)
LDPEOrgMtBO20	33.1	168.6 (15.6)	11.2 (0.8)	40.8 (4.3)
LDPEOrgMtBO40	33.5	182.2 (14.3)	11.7 (0.7)	37.5 (3.8)
LDPEOrgMtBO80	33.5	191.2 (13.8)	12.6 (0.6)	36.5 (4.2)

**Table 2:** Mean (standard deviation) values of water/oxygen barrier properties and % di(phenyl)-(2,4,6-trinitrophenyl) iminoazanium (DPPH) oxidation after 24 h of all LDPENaMtEO and LDPEOrgMtEO nanocomposite films, as well as of the LDPENaMt, LDPEOrgMt, and the pure LDPE film.

Sample Code Name	WVTR (St.Dev.) (g/h.m^2^)	OP (St.Dev.) (ccO_2_.mm/m^2^.day)	% Antioxidant Activity (St.Dev.)
LDPE	3.19 (0.40)	186.5 (3.7)	-
LDPENaMt	3.82 (0.39)	194.2 (2.6)	8.2 (2.3)
LDPENaMtTO20	3.63 (0.38)	188.4 (4.2)	51.4 (3.8)
LDPENaMtTO40	3.72 (0.41)	184.7 (6.4)	60.1 (4.1)
LDPENaMtTO80	4.88 (0.36)	192.5 (7.5)	79.8 (3.9)
LDPENaMtOO20	3.56 (0.38)	184.2 (2.8)	53.8 (3.7)
LDPENaMtOO40	3.71 (0.40)	186.8 (4.4)	61.4 (3.9)
LDPENaMtOO80	4.92 (0.20)	199.6 (6.8)	76.7 (4.2)
LDPENaMtBO20	3.62 (0.42)	191.2 (2.7)	37.9 (3.8)
LDPENaMtBO40	3.72 (0.41)	187.8 (4.3)	47.8 (3.8)
LDPENaMtBO80	4.95 (0.41)	196.6 (6.8)	55.5 (4.2)
LDPEOrgMt	2.65 (0.22)	183 (4.2)	12.0 (2.5)
LDPEOrgMtTO20	2.55 (0.20)	165 (6.4)	53.1 (4.7)
LDPEOrgMtTO40	2.50 (0.21)	155 (5.5)	58.5 (4.8)
LDPEOrgMtTO80	2.41 (0.23)	131 (6.4)	73.6 (4.9)
LDPEOrgMtOO20	2.52 (0.19)	168 (6.6)	56.0 (5.4)
LDPEOrgMtOO40	2.51 (0.20)	159 (5.2)	58.6 (5.3)
LDPEOrgMtOO80	2.21 (0.21)	136 (6.1)	73.2 (5.1)
LDPEOrgMtBO20	2.68 (0.21)	175 (6.6)	42.1 (5.0)
LDPEOrgMtBO40	2.48 (0.23)	164 (5.3)	46.1 (5.1)
LDPEOrgMtBO80	2.32 (0.21)	147 (6.7)	51.7 (5.3)

**Table 3:** Mean values’ inequality test of modulus of elasticity (E), tensile strength (σ_uts_), % elongation at break (ε_b_), water vapor transmission rate (WVTR), oxygen permeability (OP), and antioxidant activity (% Antioxidant Activity) of all tested films.

	Sig	IA
**E**	0.002	96
**σ_uts_**	0.005	90
**%** **ε_b_**	0.003	94
**WVTR**	0.003	94
**OP**	0.004	92
**%C_t,DPPH_/C_0,DPPH_**	0.001	98
